# Selective biosorption of thorium (IV) from aqueous solutions by ginkgo leaf

**DOI:** 10.1371/journal.pone.0193659

**Published:** 2018-03-06

**Authors:** Yaoyao Huang, Yang Hu, Lvcun Chen, Tao Yang, Hanfang Huang, Runping Shi, Peng Lu, Chenghua Zhong

**Affiliations:** 1 College of Environment and Resources, Chongqing Technology and Business University, Chongqing, China; 2 College of Urban Construction and Environmental Engineering, Chongqing University, Chongqing, China; Los Alamos National Laboratory, UNITED STATES

## Abstract

Low–cost biosorbents (ginkgo leaf, osmanthus leaf, banyan leaf, magnolia leaf, holly leaf, walnut shell, and grapefruit peel) were evaluated in the simultaneous removal of La^3+^, Ce^3+^, Pr^3+^, Nd^3+^, Sm^3+^, Eu^3+^, Gd^3+^, Yb^3+^, Lu^3+^, UO_2_^2+^, Th^4+^, Y^3+^, Co^2+^, Zn^2+^, Ni^2+^, and Sr^2+^ from aqueous solutions. In single metal systems, all adsorbents exhibited good to excellent adsorption capacities toward lanthanides and actinides. In a simulated multicomponent mixed solution study, higher selectivity and efficiency were observed for Th^4+^ over other metal cations, with ginkgo leaves providing the highest adsorptivity (81.2%) among the seven biosorbents. Through optimization studies, the selectivity of Th^4+^ biosorption on ginkgo leaf was found to be highly pH–dependent, with optimum Th^4+^ removal observed at pH 4. Th^4+^ adsorption was found to proceed rapidly with an equilibrium time of 120 min and conform to pseudo–second–order kinetics. The Langmuir isotherm model best described Th^4+^ biosorption, with a maximum monolayer adsorption capacity of 103.8 mg g^–1^. Thermodynamic calculations indicated that Th^4+^ biosorption was spontaneous and endothermic. Furthermore, the physical and chemical properties of the adsorbent were determined by scanning electron microscopy, Brunauer–Emmett–Teller, X-ray powder diffraction, and Fourier transform infrared analysis. The biosorption of Th from a real sample (monazite mineral) was studied and an efficiency of 90.4% was achieved from nitric acid at pH 4 using ginkgo leaves.

## Introduction

Thorium (Th) is a representative actinide metal element that is usually found alongside lanthanides and other transition metals in rocks and soil [1–5]. For instance, the mineral monazite, the main mineral source of Th^4+^, is also associated with lanthanum, cerium, samarium, yttrium, and other elements [6, 7]. Recently, the scientific importance and commercial value of Th has received much research attention because of its extensive use in various areas, including optics, radios, aeronautics, aerospace, metallurgy, chemical and nuclear industries, materials science [8–12], and nuclear medicine. In particular, Th is considered a promising (neutron irradiation) fertile material for producing nuclear fuel because it is associated with fewer radioactive fission products and produces lower quantities of highly radiotoxic actinides [13–16]. However, excessive amounts of Th have entered the environment due to these activities [17, 18]. Th released into the environment can reach the top of the food chain and be ingested by humans, causing irreversible damage to multiple organs and even death [19–22]. Therefore, the removal of Th ions from aqueous media is vital due to the detrimental effects to biological systems associated with its radioactivity and toxicity [23].

Many techniques have been used for Th separation, including precipitation [24–26], solvent extraction [27, 28], and adsorption [9, 21, 29–35]. Among these processes, adsorption is arguably the simplest, most adaptable, useful, and well–established technique for Th separation [36]. However, some of the adsorbents used remain costly, meaning that agricultural and forestry by–products could be effectively exploited as inexpensive biosorbents for Th removal [17, 19, 37–40]. Agriculture and forestry wastes are the richest sources of low–cost biosorbents [41, 42]. Among them, cellulose, hemicellulose, pectin (galacturonic acid), and lignin acid bear various polar functional groups, including carboxylic and phenolic acid groups, which can take part in Th–ion complexation [43]. A few examples of biosorbents able to separate Th from other metal ions have been reported, including rice hulls [40], wheat bran [44], and olive cake [39].

Ginkgo, one of the oldest living plant species, is often referred to as “a living fossil” [45, 46]. Ginkgo leaves are similar to many peels, but boast a large flavonoid content that provides a plentiful source of carbonyl groups to bind with metals [47]. Despite previous research on mercury (Hg) pollution [48] and chromium (Cr) ion sorption [49] involving ginkgo leaves, the adsorption of other metal ions has been paid little attention, and no studies have applied ginkgo leaves to Th separation. In this work, ginkgo leaves and six other common biosorbents, namely, osmanthus leaves, banyan leaves, magnolia leaves, holly leaves, walnut shells, and grapefruit peel, were chosen to study their selectivity for separating Th from mixed solutions containing representative lanthanides, actinides, and other metal cations in a fast and facile room–temperature procedure. The results showed that selective Th^4+^ adsorption from aqueous solution was higher for ginkgo leaves than for the other six biosorbents. To our knowledge, this work represents the first example of employing ginkgo leaves for the selective biosorption of lanthanides and actinides, specifically Th.

## Materials and methods

### Reagents

Unless stated otherwise, all chemicals were of analytical grade. Lanthanum nitrate hexahydrate (La(NO_3_)_3_·6H_2_O), praseodymium nitrate hexahydrate (Pr(NO_3_)_3_·6H_2_O), neodymium nitrate hexahydrate (Nd(NO_3_)_3_·6H_2_O), samarium nitrate hexahydrate (Sm(NO_3_)_3_·6H_2_O), europium nitrate hexahydrate (Eu(NO_3_)_3_·6H_2_O), gadolinium nitrate hexahydrate (Gd(NO_3_)_3_·6H_2_O), and ytterbium nitrate pentahydrate (Yb(NO_3_)_3_·5H_2_O) were purchased from the Aladdin Reagent Co., Ltd. (Shanghai, China). Lutetium nitrate hexahydrate (Lu(NO_3_)_3_·6H_2_O), thorium nitrate hydrate (Th(NO_3_)_4_·4H_2_O), uranyl nitrate hydrate (UO_2_(NO_3_)_2_·6H_2_O), zinc nitrate hexahydrate (Zn(NO_3_)_2_·6H_2_O), and nickel nitrate hexahydrate (Ni(NO_3_)_2_·6H_2_O) were obtained from Chu Shengwei Chemical Co., Ltd (Hubei, China). Cerium nitrate hexahydrate (Ce(NO_3_)_3_·6H_2_O), cobalt nitrate hexahydrate (Co(NO_3_)_2_·6H_2_O), yttrium nitrate hexahydrate (Y(NO_3_)_3_·6H_2_O), nitric acid (HNO_3_), and sodium hydroxide (NaOH) were obtained from Chengdu Kelong Chemical Factory. Strontium nitrate (Sr(NO_3_)_2_) was obtained from Guangdong Guanghua Sci-Tech Co., Ltd. China. Stock solutions of metal ions (1000 mg L^–1^) were prepared by dissolving the appropriate amounts of La(NO_3_)_3_·6H_2_O, Ce(NO_3_)_3_·6H_2_O, Pr(NO_3_)_3_·6H_2_O, Nd(NO_3_)_3_·6H_2_O, Sm(NO_3_)_3_·6H_2_O, Eu(NO_3_)_3_·6H_2_O, Gd(NO_3_)_3_·6H_2_O, Yb(NO_3_)_3_·5H_2_O, Lu(NO_3_)_3_·6H_2_O, Th(NO_3_)_4_·4H_2_O, UO_2_(NO_3_)_2_·6H_2_O, Co(NO_3_)_2_·6H_2_O, Zn(NO_3_)_2_·6H_2_O, Sr(NO_3_)_2_, Ni(NO_3_)_2_·6H_2_O, and Y(NO_3_)_3_·6H_2_O in pH 4 nitric acid solution (100 mL). Working solutions were prepared daily by diluting the stock solution with dilute nitric acid.

### Instruments and apparatus

All metal ions were determined using inductively coupled plasma optical emission spectrometry (ICP–OES). Shaking was applied using a SHA–C water bath oscillator and a PHS–3C pH meter for regulating pH. The exterior surfaces of ginkgo leaf powder (GLP), osmanthus leaf powder (OLP), banyan leaf powder (BLP), magnolia leaf powder (MLP), holly leaf powder (HLP), walnut shell powder (WSP), and grapefruit peel powder (GFP) were observed using scanning electron microscopy (SEM; JEOL SU–8010, Japan). X–ray diffraction (XRD) was used to determine the mineralogy of the samples (Shimadzu 6100, Japan). The specific surface area and pore structure of the samples were determined using N_2_ adsorption–desorption isotherms (Micromeritics, ASAP 2020, USA). Fourier transform infrared (FTIR) spectra were recorded on a Prestige–21 FTIR spectrometer to confirm changes in functional groups on the biosorbent surface before and after Th^4+^ biosorption. All glassware was soaked in 5% HNO_3_ for 24 h and rinsed with distilled water before and after use to remove trace metal ions.

### Preparation of biosorbents

Ginkgo leaf, osmanthus leaf, banyan leaf, magnolia leaf, and holly leaf used in this study were obtained from the campus of Chongqing Technology and Business University. Walnut shell was obtained from thin-skinned walnuts in Hebei Province, while grapefruit peel was obtained from Guanxi grapefruits from Fujian Province. The biomaterials were prepared by washing several times with water and once with distilled water, drying to a constant weight at 65°C, and grinding to a powder. The obtained ginkgo leaf powder (GLP), osmanthus leaf powder (OLP), banyan leaf powder (BLP), magnolia leaf powder (MLP), holly leaf powder (HLP), walnut shell powder (WSP), and grapefruit peel powder (GFP) were sieved into fractions, using 40, 100, 150, 200, 300 mesh standard test sieves to obtain the corresponding particle sizes. The sieved powders were stored in a desiccator until used.

### Experimental procedures

Batch experiments were performed at room temperature (25 ± 1°C) and conducted under shaking conditions (120 rpm) in 50–mL capped tubes. The cation solution (20 mL) of known concentration (100 mg L^–1^) was adjusted to the desired pH by adding sodium hydroxide or nitric acid, followed by a known amount of biosorbent (0.04 g). The tubes were then mixed thoroughly. After allowing sufficient time for adsorption equilibrium to be reached, the biosorbent was separated by filtration (filter paper). The metal ion concentration in the filtrate was then determined by ICP–OES. To obtain accurate adsorption results, three independent experiments were performed and the average percentage of metal adsorbed was calculated. scanning electron microscopy (SEM), Brunauer–Emmett–Teller (BET), XRD, and FTIR analysis were performed on biosorbents before and after metal ion sorption.

The adsorption efficiency of the biosorbent with respect to the adsorbed metal ions, R (%), was calculated using Eq (1) [44]:

$$R(\%)=(C_{0}-{C_{e}/C}_{0})\times 100$$ (1)

where C_0_ and C_e_ are the initial and equilibrium concentrations (mg L^–1^) of analyte ions in the solution, respectively. The amount of metal ions adsorbed onto the biosorbent (q_sorp_, mg g^–1^) was calculated using Eq (2) [50]:

qsorp=(C0−Ce)Vm (2)

where C_0_ and C_e_ are the initial and equilibrium concentrations (mg L^–1^) of analyte ions in the solution, respectively, m is the biosorbent weight (g), and V is the sample solution volume (L).

### Method for thorium extraction from monazite minerals

Monazite is an important commercial source of thorium. Monazite samples were obtained from Lingshou County jade product processing plant and sieved to a mesh size of 1250. Monazite powder (600 mg) was digested with dilute nitric acid solution (pH 1) in a beaker with magnetic stirring. After stirring for 12 h and filtering, the filtrate was adjusted to pH 4 and diluted to a final volume of 250 mL for use in experiments. The filtrate (20 mL, pH 4) was contacted with biosorbent (0.04 g). After shaking for 120 min at 120 r/min, the sorbent was filtered. Metal ion concentrations in the filtrate were determined directly by ICP-OES. Metal ions sorbed on the biomass, R (%), were calculated using the equation shown in the experimental procedures. Experiments were performed in triplicate for each biosorbent.

## Results and discussion

### Selection of effective Th^4+^ biosorbent

**Fig 1:**
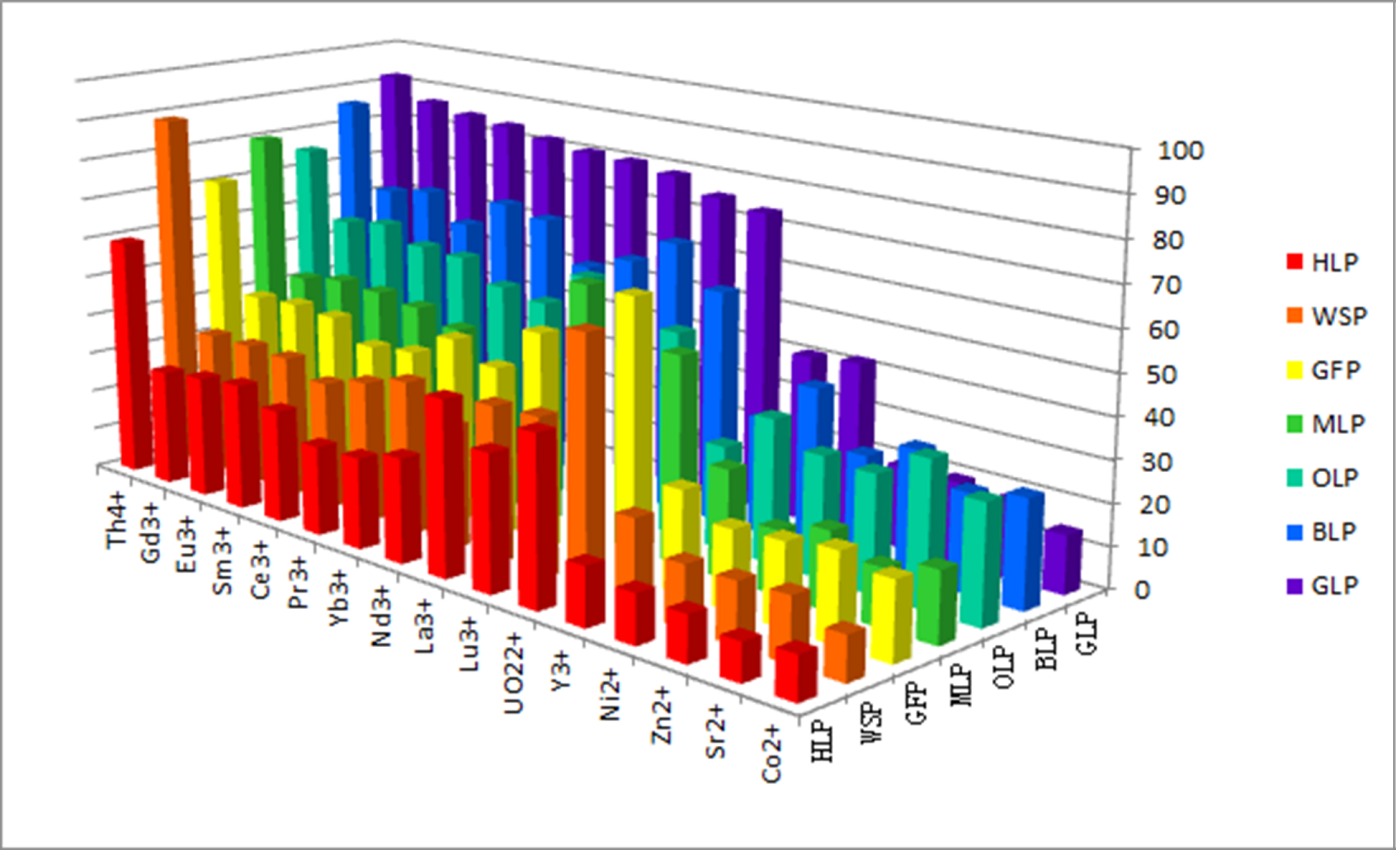
Biosorption efficiencies [% on solid phase] of GLP, OLP, BLP, MLP, HLP, WSP, and GFP biosorbents in individual metal ion solutions (pH 4; contact time, 120 min; biosorbent concentration, 2 g L^–1^; initial metal ion concentration, 100 mg L^–1^; temperature, 298 K).

**Fig 2:**
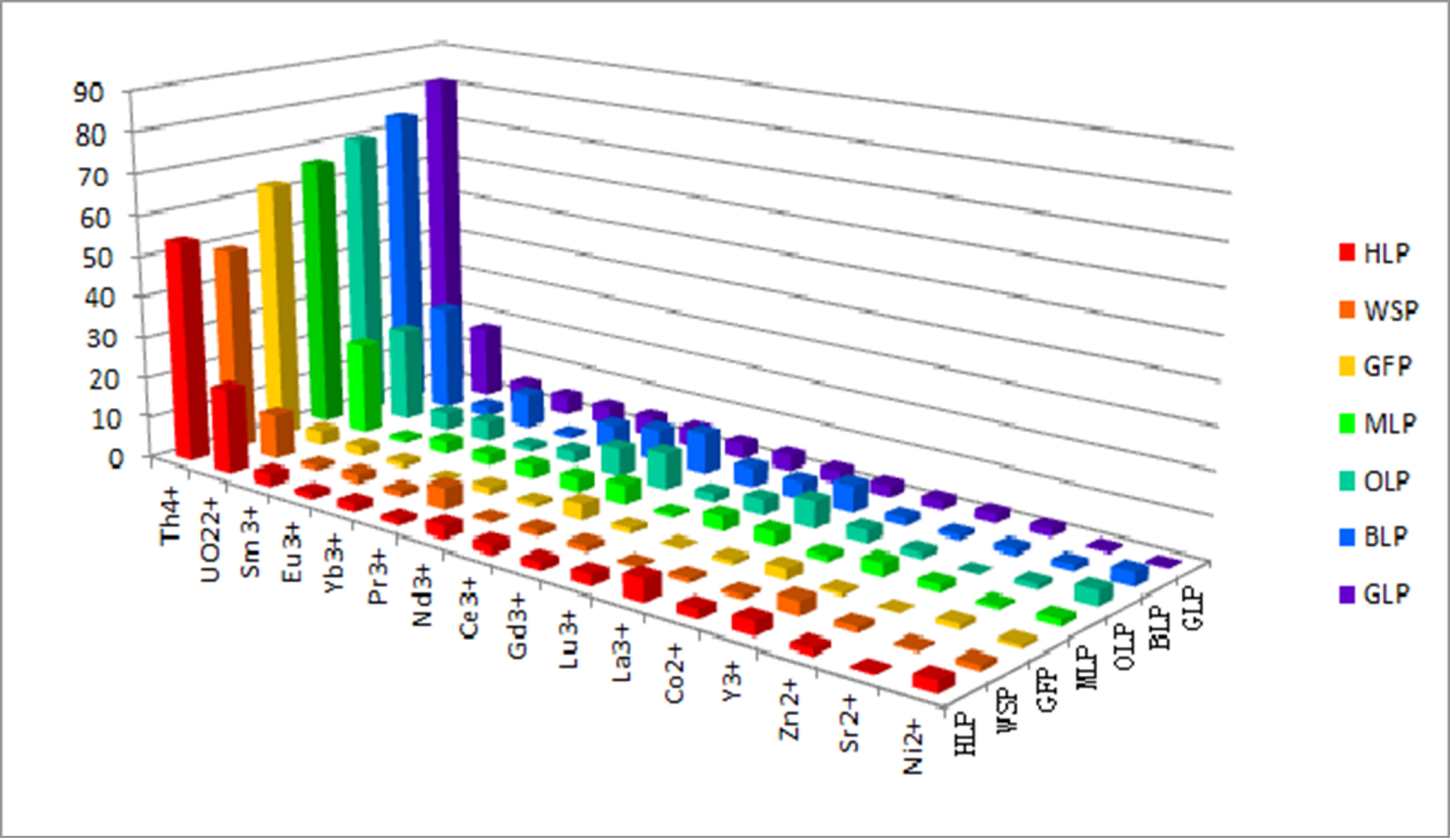
Biosorption efficiencies [% on solid phase] of GLP, OLP, BLP, MLP, HLP, WSP and GFP biosorbents in a mixed solution containing different metal ions (pH 4; contact time, 120 min; biosorbent concentration, 2 g L^–1^; initial metal ion concentration, 100 mg L^–1^; temperature, 298 K).

The adsorption efficiencies of GLP, OLP, BLP, MLP, HLP, WSP, and GFP for sixteen metal nitrates, including nine lanthanides (La^3+^, Ce^3+^, Pr^3+^, Nd^3+^, Sm^3+^, Eu^3+^, Gd^3+^, Yb^3+^, and Lu^3+^), two actinides (UO_2_^2+^ and Th^4+^), four transition metal ions (Y^3+^, Co^2+^, Zn^2+^, and Ni^2+^) and an alkali earth metal (Sr^2+^), were measured. Biosorption percentages are shown in Table 1 and Fig 1. All biosorbents exhibited good to excellent adsorption capacities towards the lanthanides, Th^4+^, and UO_2_^2+^. For example, the adsorption efficiencies for Th^4+^ were 92.0% for GLP, 75.3% for OLP, 86.0% for BLP, 80.1% for MLP, 59.4% for HLP, 88.7% for WSP, and 70.9% for GFP, while the adsorptivity of transition metal ions and Sr^2+^ were markedly lower, at 14.0–42.0% for GLP, 28.2–34.9% for OLP, 23.7–38.9% for BLP, 13.8–18.6% for MLP, 9.1–14.2% for HLP, 10.6–21.5% for WSP, and 18.7–24.5% for GFP. Despite the successful biosorption of individual metal ions under controlled conditions, these efficiencies may differ in solutions containing multiple metal ions. Therefore, to test the potential selectivity for Th^4+^ adsorption among competing ions, a multicomponent mixed solution containing Th^4+^ and 15 other types of metal ion was prepared. The results are shown in Table 2 and Fig 2.

**Table 1:** Adsorption efficiencies [% on solid phase] of GLP, OLP, BLP, MLP, HLP, WSP, and GFP for metal ions in individual aqueous metal nitrate solutions.

Metal ion	Biosorption^a^						
	GLP	OLP	BLP	MLP	HLP	WSP	GFP
Th^4+^	92.0±0.6	75.3±0.5	86.0±0.3	80.1±0.8	59.4±1.0	88.7±1.0	70.9±0.8
Gd^3+^	86.5±1.1	58.3±0.3	64.5±0.5	45.5±0.9	28.5±0.2	35.4±0.8	42.9±0.8
Eu^3+^	84.7±1.1	59.8±0.7	66.3±0.7	47.3±0.6	29.8±0.2	35.4±2.3	43.5±1.2
Sm^3+^	83.7±0.2	56.2±1.3	59.8±1.2	46.7±1.5	30.9±0.7	35.0±1.9	42.8±0.9
Ce^3+^	81.9±0.3	55.7±1.2	67.2±0.6	45.3±0.3	27.4±1.3	31.3±0.9	37.9±0.9
Pr^3+^	80.5±0.5	50.5±0.5	65.3±0.8	42.3±1.8	21.7±0.7	34.4±0.9	39.2±1.2
Yb^3+^	80.0±1.1	48.9±1.3	55.6±1.5	34.2±1.6	22.1±1.5	37.5±0.3	45.2±0.7
Nd^3+^	78.7±0.4	57.8±0.7	59.5±0.2	40.1±1.5	25.3±0.8	29.8±0.7	41.0±0.6
La^3+^	75.2±1.9	42.1±0.5	66.1±1.4	61.0±1.6	42.3±0.5	37.7±0.7	52.0±1.4
Lu^3+^	73.6±1.7	49.5±1.1	56.7±1.3	46.0±0.1	33.0±1.4	38.2±0.4	41.8±1.0
UO_2_^2+^	40.8±0.5	24.9±1.5	28.4±1.1	49.6±1.4	40.7±0.5	60.3±1.5	65.8±1.3
Y^3+^	42.0±1.1	34.6±1.7	38.9±0.8	25.9±1.8	14.2±1.1	21.5±1.2	24.5±1.1
Ni^2+^	19.8±1.0	29.1±1.2	25.9±1.3	15.2±1.3	11.9±1.8	14.6±1.6	18.9±0.8
Zn^2+^	19.5±1.4	28.2±0.4	30.5±1.5	18.6±2.1	11.1±1.0	14.6±1.2	19.7±1.1
Sr^2+^	18.0±0.5	34.9±1.0	23.7±2.3	13.8±0.8	9.1±1.3	15.2±1.1	21.3±0.3
Co^2+^	14.0±0.1	28.5±0.1	26.0±1.4	17.0±1.2	10.4±0.8	10.6±0.8	18.7±0.5

^**a**^Average values for three independent adsorption experiments; precision corresponds to ± σ_n-1_, where σ_n-1_ is the standard deviation of the mean.

**Table 2:** Adsorption efficiencies [% on solid phase] of GLP, OLP, BLP, MLP, HLP, WSP, and GFP for aqueous metal nitrates in mixed solution.

Metal ion	Biosorption^a^						
	GLP	OLP	BLP	MLP	HLP	WSP	GFP
Th^4+^	81.2±1.7	70.3±1.0	74.0±0.8	66.3±1.4	53.9±0.7	49.1±2.5	62.7±1.0
UO_2_^2+^	17.5±1.8	23.0±0.9	25.5±0.6	22.2±0.8	20.7±0.6	10.8±1.0	3.5±0.4
Sm^3+^	5.2±0.3	4.1±0.7	2.6±0.4	1.0±0.2	2.8±0.5	1.0±0.4	2.4±0.3
Eu^3+^	4.9±0.7	1.3±0.4	8.6±0.9	3.2±0.4	1.6±0.4	2.1±0.2	1.3±0.4
Yb^3+^	4.7±0.4	1.4±0.5	1.0±0.5	2.7±0.9	2.2±0.4	1.5±0.2	0.1±0.0
Pr^3+^	4.7±0.4	3.0±0.6	6.0±0.3	2.9±0.8	1.4±0.3	5.3±0.9	1.7±0.3
Nd^3+^	4.6±0.2	6.7±1.0	7.9±0.4	3.3±0.5	3.3±0.8	0.6±0.6	1.1±0.1
Ce^3+^	3.9±0.3	8.4±0.9	9.9±0.7	4.8±0.2	2.3±0.2	1.4±0.8	4.0±0.8
Gd^3+^	3.8±0.9	2.2±0.6	4.4±0.4	0.8±0.2	2.0±0.7	1.4±0.1	1.3±0.3
Lu^3+^	3.0±1.7	3.9±0.7	4.2±0.5	3.6±0.6	2.7±0.4	0.5±0.2	0.3±0.2
La^3+^	2.8±0.2	6.7±0.4	6.6±0.8	3.2±0.3	5.3±0.6	1.1±0.6	1.0±0.2
Co^2+^	2.5±0.4	3.0±0.3	2.0±0.6	1.4±0.2	2.3±0.2	0.9±0.2	2.7±0.2
Y^3+^	2.4±0.6	1.4±0.4	1.2±0.3	3.3±0.3	3.4±0.3	3.4±0.7	0.7±0.0
Zn^2+^	2.0±1.1	1.4±0.3	1.5±0.5	1.9±0.1	1.4±0.4	1.4±0.2	0.1±0.0
Sr^2+^	0.5±0.2	1.3±0.2	1.6±0.3	0.8±0.2	0.4±0.1	0.5±0.0	1.3±0.3
Ni^2+^	0.3±0.1	3.5±0.3	3.4±0.3	1.6±0.1	2.8±0.4	1.3±0.3	0.7±0.1

^**a**^Average values for three independent adsorption experiments; precision corresponds to ± σ_n-1_, where σ_n-1_ is the standard deviation of the mean.

The results showed that the adsorptivities of all cations were lower in the mixed solution than in the single–metal solutions. However, the adsorption efficiency for Th ions remained higher than that of other metal cations. Notably, GLP showed an adsorptivity of 0.3–5.2% for most metal ions, except for Th^4+^ and UO_2_^2+^. A moderate adsorptivity of 17.5% was obtained for UO_2_^2+^, while Th^4+^ was efficiently adsorbed with the highest R (%) value of 81.2%. This indicated that GLP was more selective for Th^4+^ adsorption than for that of other metal cations. For OLP, BLP, MLP, HLP, WSP, and GFP, similar lower adsorption efficiencies were observed for almost all metal cations examined. The R (%) values for Sm^3+^, Eu^3+^, Yb^3+^, Pr^3+^, Nd^3+^, Ce^3+^, Gd^3+^, Lu^3+^, La^3+^, Co^2+^, Y^3+^, Zn^2+^, Sr^2+^, Ni^2+^, and UO_2_^2+^ were the range of 1.3–23.0% for OLP, 1.0–25.5% for BLP, 0.8–22.2% for MLP, 0.4–20.7% for HLP, 0.5–10.8% for WSP, and 0.1–4.0% for GFP. The exception was Th^4+^ adsorption, which presented much higher adsorptivities than other cations, of 49.1% on WSP and 62.7% on GFP. Therefore, for all seven biosorbents, the selectivity for was highest for Th among all metal cations tested. This might be due to Th^4+^ being tetravalent, which results in a stronger electrostatic force toward the carbonyl groups in the biosorbents than for trivalent cations [51]. Therefore, GLP is a promising candidate for the recovery and separation of Th^4+^ from mixtures of actinides, lanthanides, and other metal ions.

**Fig 3:**
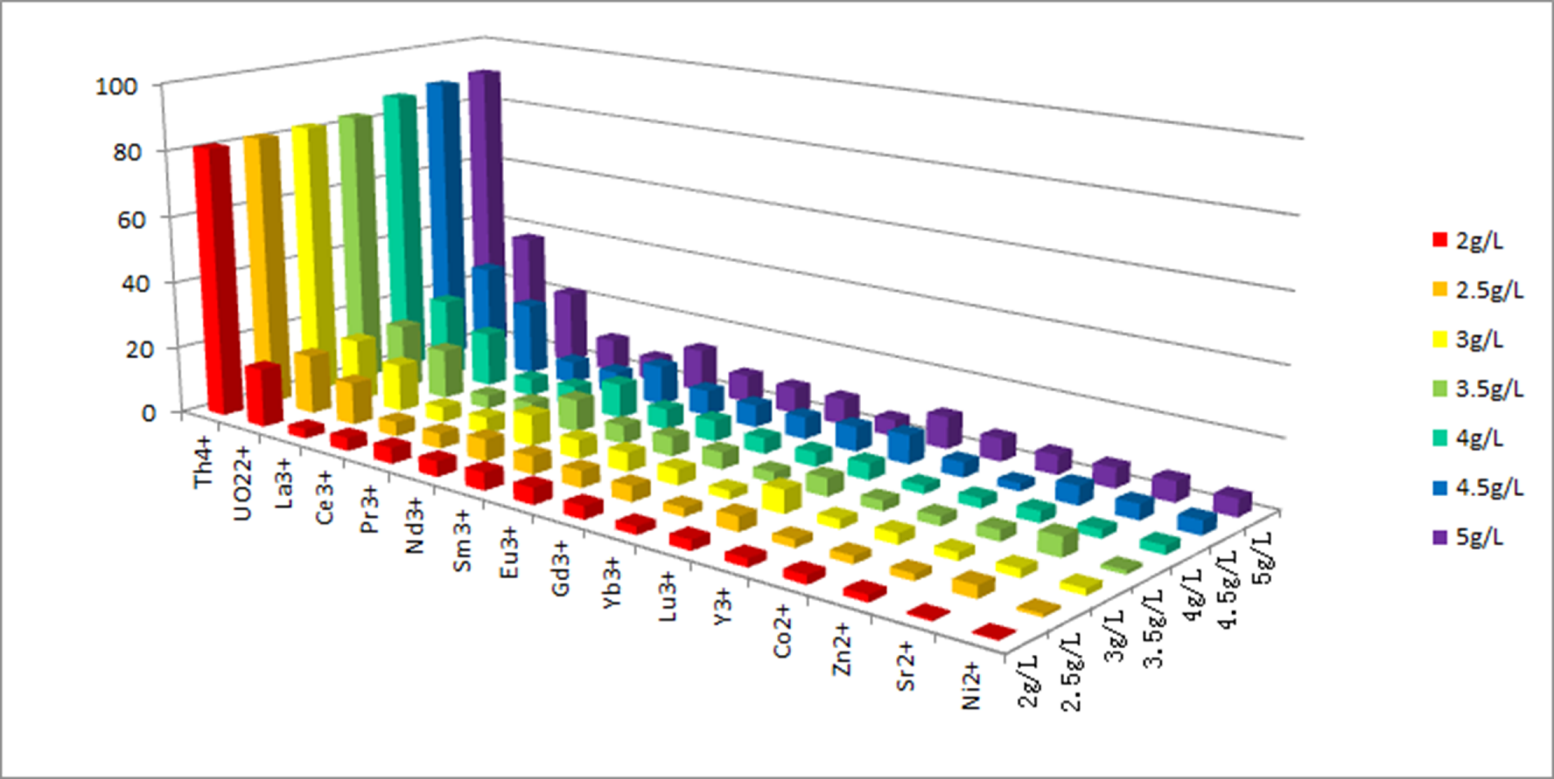
Effect of different amounts of adsorbent (GLP) on the removal of each element from a mixed solution (pH 4; contact time, 120 min; initial metal ion concentration, 100 mg L^–1^; temperature, 298 K).

To assess the adsorption capacity of GLP toward metal ions, various nitrate metal salts were screened in a mixed aqueous medium using the same adsorption technique. The results are shown in Fig 3.

Further intriguing results were obtained using different GLP concentrations for the biosorption of mixed aqueous ions, as shown in Fig 3. With an increasing amount of GLP, the adsorption efficiency of each ion increased. Notably, the adsorption efficiency of Th ions was much higher than that of other ions at each GLP concentration. When the biosorbent concentration was 5 g L^–1^, the removal rate reached more than 90%. This demonstrated that GLP had high Th^4+^ selectivity in the presence of competing ions and, therefore, some potential for practical application. However, the origin of this selectivity is not yet fully understood. The preferential biosorption of Th^4+^ over other metal ions by GLP might be due to the rich flavonoid content of GLP, which endows GLP with a multitude of carbonyl groups that provide a coordination site that binds highly–charged Th^4+^ better than other metal ions [52].

Having selected GLP for further experiments, the effects of various experimental parameters (pH, contact time, biosorbent concentration, particle size, initial Th concentration, and temperature) on adsorption were investigated using a “one variable at a time” method [40].

### Optimization of experimental parameters for improved Th^4+^ adsorption efficiency

#### Effect of solution pH

**Fig 4:**
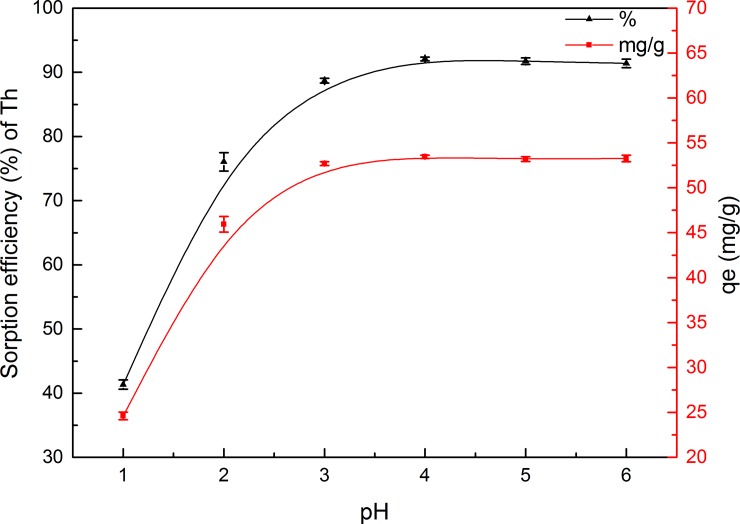
Effect of pH on Th adsorption onto GLP (contact time, 120 min; biosorbent concentration, 2 g L^–1^; particle size, 150–200 mesh; initial metal ion concentration, 100 mg L^–1^; temperature, 298 K).

Among all other parameters, the aqueous solution pH is the most important, as it effects both metal ion speciation and the charge at adsorption sites in the biological adsorbents [53, 54]. Accordingly, this work aimed to evaluate Th^4+^ biosorption from aqueous solution by GLP and determine changes in uptake levels in the pH range 1–6. Fig 4 shows the effect of initial pH on Th^4+^ biosorption.

The results showed that, in general, the adsorption efficiency increased steadily with increasing pH (Fig 4). At low pH values, adsorption usually occurred with a low removal efficiency. Th removal increased by about 50.8% with increasing pH, (from 41.3% at pH 1 to 92.1% at pH 4), but decreased again slightly at pH > 4. Furthermore, the biosorption capacity significantly increased from 24.6 to 53.5 mg g^–1^ with an increase in pH from 1 to 4, and remained almost constant above pH 4. This trend in biosorption was due to competition at binding sites between hydrogen ions and Th ions [40]. At low pH, surface ligates were closely associated with hydronium ions (H_3_O^+^), which restricted metal cation approach through electrostatic repulsion [55, 56]. At higher pH values, fewer H^+^ ions and the increase in negatively charged ligand sites resulted in improved metal ion biosorption [57]. Based on these results, pH 4 was selected as the optimum pH for subsequent experiments.

#### Effect of contact time

**Fig 5:**
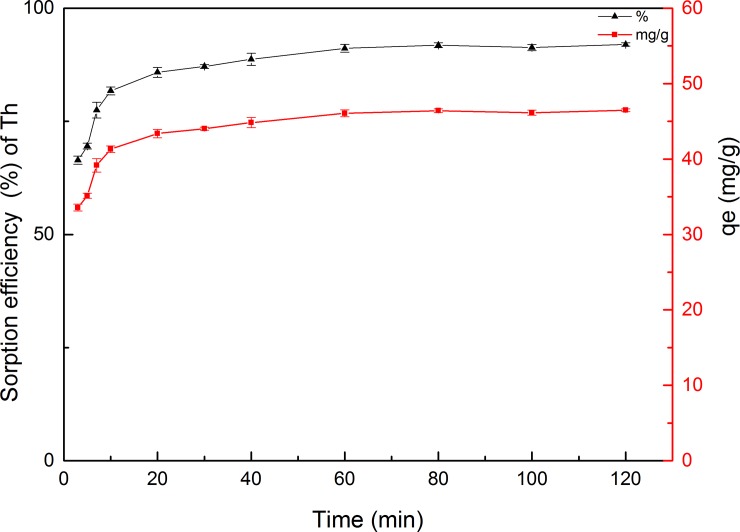
Time–dependence of Th adsorption on GLP (pH 4; biosorbent concentration, 2 g L^–1^; particle size, 150–200 mesh; initial metal ion concentration, 100 mg L^–1^; temperature, 298 K).

Contact time is another important parameter for the successful application of biosorbents in practical and rapid adsorption [58]. The time profiles of Th adsorption on GLP with respect to percentage adsorption and adsorption capacity are shown in Fig 5. The sorption efficiency was relatively high, with more than 80% adsorption reached within 10 min, while the aqueous solution and GLP established distribution equilibria in less than 80 min. Consequently, the contact time was increased to 120 min in subsequent trials to ensure that an equilibrium was present between the solution and biosorbent. Therefore, an initial rapid phase occurs before equilibrium is slowly achieved [59]. Most adsorption systems display these two phases because the sorbent initially contains a high concentration of exchangeable binding sites for metal ions. This phenomenon indicates that, with increasing contact time, these binding sites gradually become fewer until reaching saturation, which resulted in decreased uptake and the adsorption reaction reaching equilibrium.

#### Effect of adsorbent concentration

**Fig 6:**
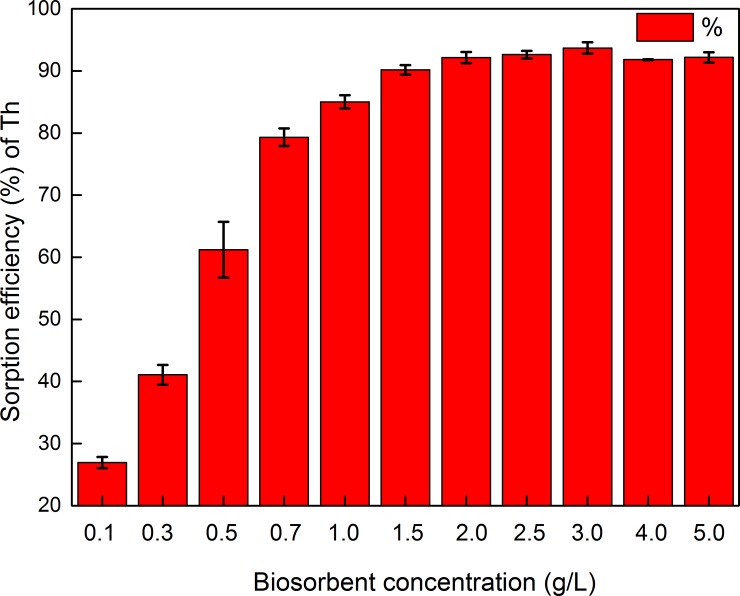
Influence of biosorbent concentration on the percentage of adsorbed Th ion. (pH 4; contact time, 120 min; particle size, 150–200 mesh; initial metal ion concentration, 100 mg L^–1^; temperature, 298 K).

Next, the amount of GLP (g L^–1^) added to the analyte solution was investigated. As shown in Fig 6, GLP concentrations ranging from 0.1 to 5 g L^–1^ were used to determine the effect of GLP concentration on the biosorption rate.

Increasing the adsorbent concentration significantly increased Th^4+^ biosorption. When the concentration was ≥2.0 g L^–1^, the Th^4+^ biosorption efficiency was already more than 90%, and showed little further increase at higher biosorbent concentrations. This trend could be due to the partial aggregation of biosorbent at higher concentrations, which would result in a lower effective surface area for biosorption and, consequently, lower Th uptake per unit mass of biosorbent [60, 61]. Therefore, 2.0 g L^–1^ was selected as the optimum biosorbent concentration for all subsequent experiments.

#### Effect of particle size

**Fig 7:**
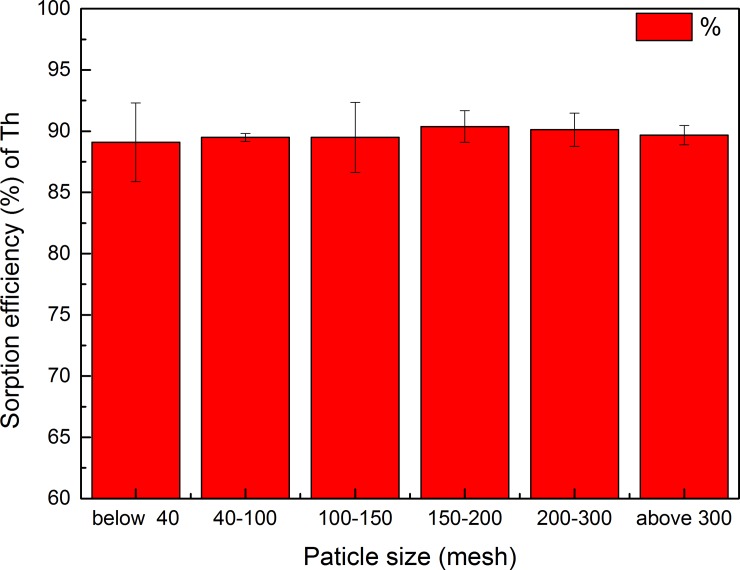
Effect of GLP particle size on uptake Th^4+^ (pH 4; contact time, 120 min; biosorbent concentration, 2 g L^–1^; initial metal ion concentration, 100 mg L^–1^; temperature, 298 K).

The GLP particle size, which is another important parameter for Th^4+^ uptake and heavily influences the cost of the process, was investigated. As shown in Fig 7, a reduction in Th^4+^ removal was observed with decreasing biosorbent particle size. An adsorbent particle size in the range 150–200 mesh resulted in slightly better adsorption compared to other particle sizes, but the size of the biosorbent particle size had little influence on adsorption.

#### Effect of initial Th^4+^ concentration

**Fig 8:**
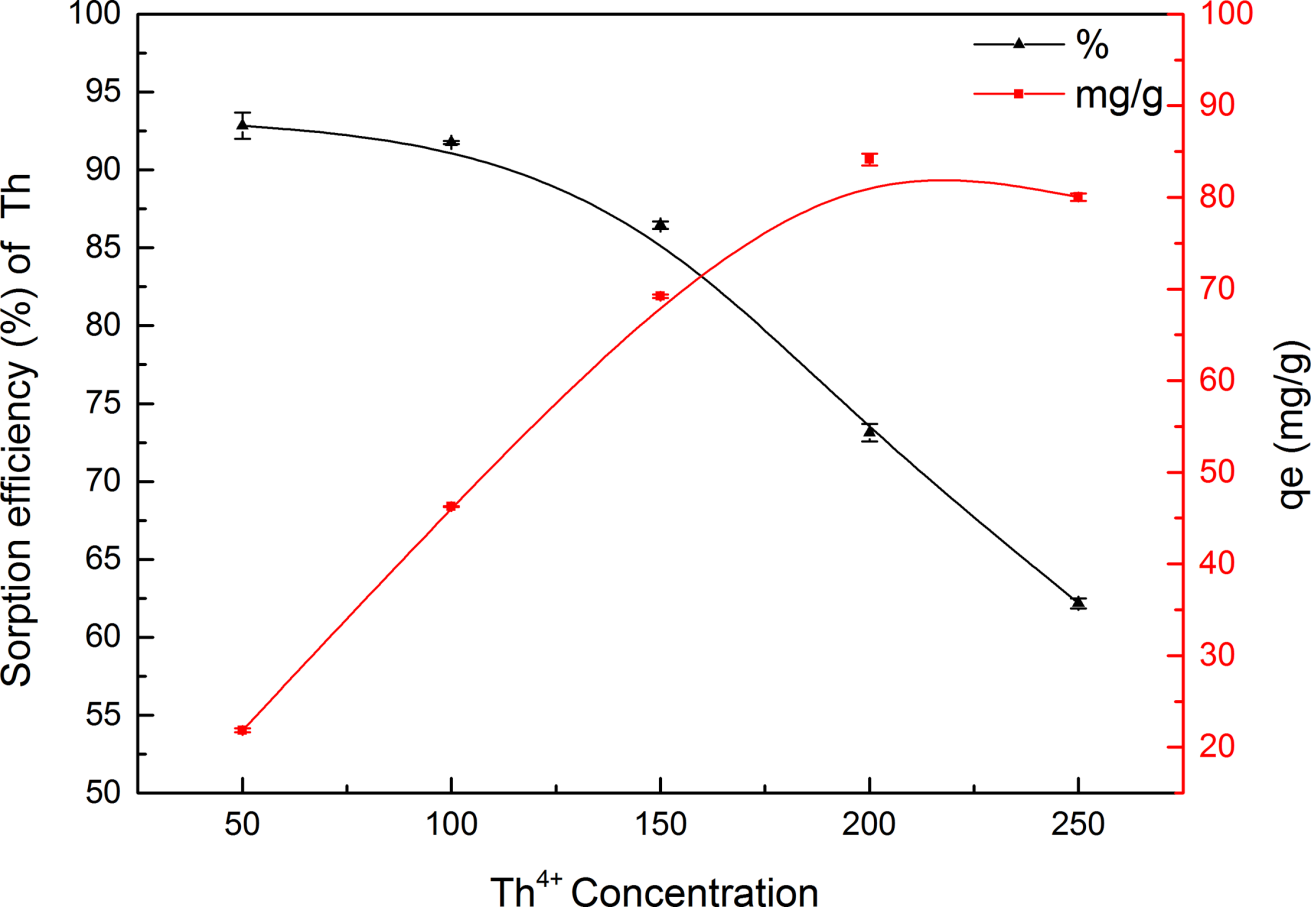
Effect of Th concentration on Th^4+^ uptake by GLP (pH 4; contact time, 120 min; biosorbent concentration, 2 g L^–1^; particle size, 150–200 mesh; temperature, 298 K).

Under optimum conditions, the effect of initial Th^4+^ concentration in the adsorption experiments was studied in the range 50–250 mg L^–1^. The maximum biosorption yield was achieved at the lowest Th concentration (50 mg L^–1^), with increasing initial Th^4+^ concentration leading to a clear decrease in adsorption efficiency from 92.8% to 62.2%, while the amount of Th^4+^ adsorbed per unit mass of GLP increased from 21.9 to 80 mg g^–1^ (Fig 8). However, a slight decrease in adsorption capacity from 84 mg g^–1^ to 80 mg g^–1^ was observed when the initial concentration was increased from 200 to 250 mg L^–1^. This change might be due to the available metal binding sites on biosorbents approaching saturation at higher Th^4+^ concentrations.

#### Effect of temperature

**Fig 9:**
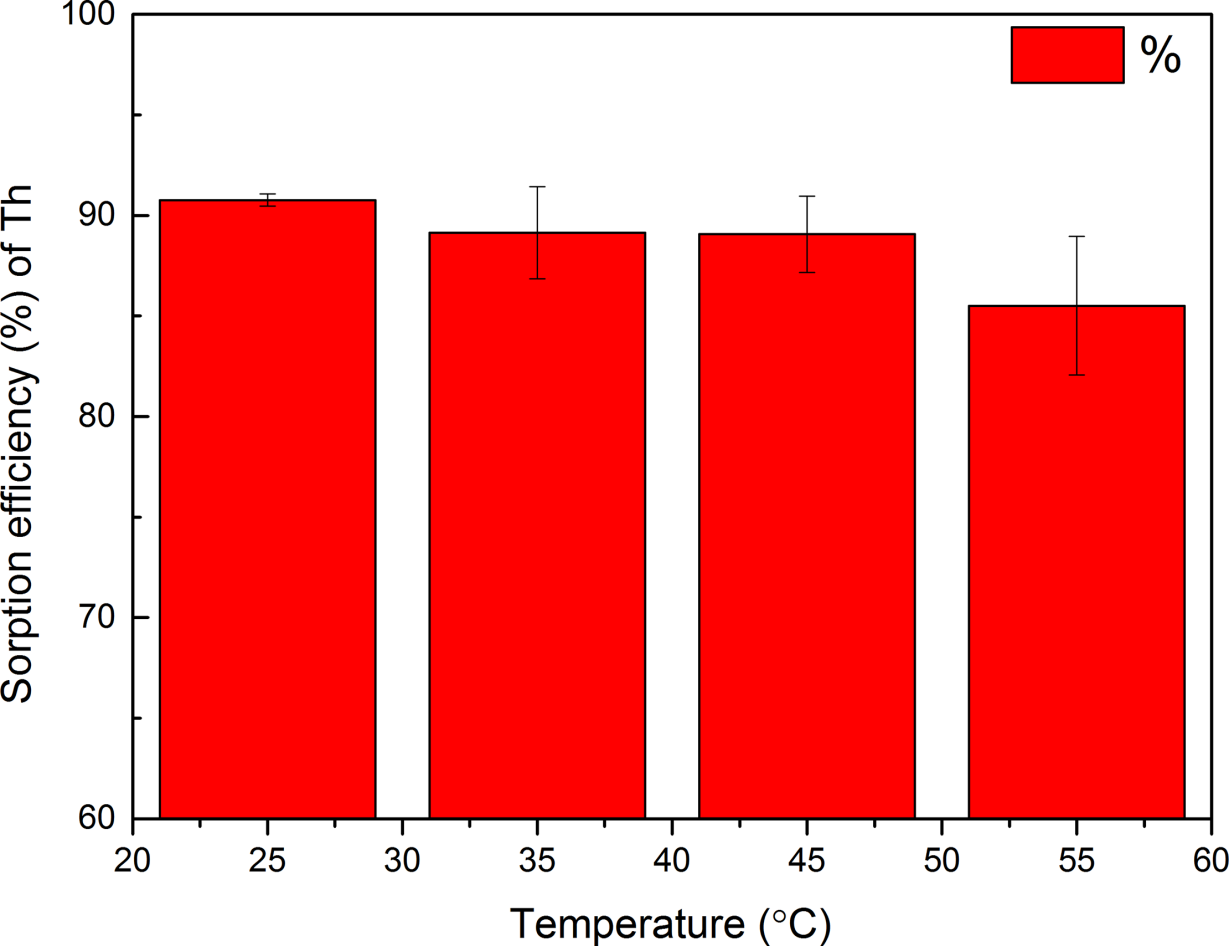
Effect of temperature on Th^4+^ uptake by GLP (pH 4; contact time, 120 min; biosorbent concentration, 2 g L^–1^; particle size, 150–200 mesh; initial metal ion concentration, 100 mg L^–1^).

To determine the effect of temperature on Th^4+^ biosorption by GLP, biosorption experiments were performed at 25–55°C (Fig 9). With increasing temperature from 25 to 55°C, the adsorption efficiency decreased from 90.8 to 85.5%, which indicated that the biosorption mechanism was energy dependent and exothermic.

### Insight into biosorption mechanism

#### Modeling of adsorption isotherms

Adsorption isotherms can be used to describe the adsorbent capacity and relationship between adsorbent and adsorbate at equilibrium, usually expressed as the ratio of quantity adsorbed to the quantity remaining in solution at equilibrium at a fixed temperature [62]. In the present study, equilibrium data was evaluated using the Langmuir and Freundlich models.

The Langmuir adsorption isotherm assumes an adsorbate monolayer covering a homogeneous adsorbent surface, with an equal adsorption activation energy for each molecule adsorbed on the surface. This model has been successfully applied to many adsorption processes, including solute adsorption from liquid solutions, for which it is the most widely used adsorption isotherm [61]. The Langmuir isotherm is commonly presented as:

$$q_{e}=\frac{q_{m}K_{L}C_{e}}{1+K_{L}C_{e}}$$ (3)

where q_m_ (mg g^–1^) is the q_e_ of a complete monolayer, which is a constant related to adsorption capacity, and K_L_ (L mg^–1^) is a constant related to binding site affinity and adsorption energy.

The Freundlich isotherm describes multilayer adsorption on a heterogeneous surface where the heat of adsorption has a nonuniform distribution [61], and is commonly presented as [63]:

$$q_{e}=K_{F}C_{e}^{1/n}$$ (4)

where K_F_ and 1/n are Freundlich constants related to the adsorption capacity and adsorption intensity of the adsorbent, respectively.

All isotherm equation parameters and R^2^ values are listed in Table 3. The results obtained showed that the adsorption characteristics of Th^4+^ on GLP followed the Langmuir isotherm equation more closely. This suggested that the metal ions were adsorbed as a monolayer on the biosorbent surface. The maximum Th^4+^ uptake capacity (q_m_) of GLP was found to be 103.8 mg g^–1^.

**Table 3:** Langmuir and Freundlich constants for Th biosorption on GLP.

Ion	Langmuir			Freundlich		
	K_L_	q_m_	R^2^	K_F_	1/n	R^2^
Th^4+^	0.08118	103.8	0.98461	17.52	0.37414	0.81471

#### Dynamic modeling

Rate–determining steps of biosorption processes are usually described using kinetics models [64]. Four widely used kinetic models, namely, pseudo–first–order and pseudo–second–order models, the Elovich equation, and the intra–particle diffusion model, were used to analyze the kinetic data and determine the rate–determining step of Th^4+^ biosorption on GLP.

The pseudo–first–order equation is expressed as [65]:

$$q_{t}=q_{e}(1-e^{-k_{1}t})$$ (5)

where q_e_ and q_t_ are the amount of solute adsorbed (mg g^–1^) at equilibrium and time t (min), respectively, and k_1_ is the pseudo–first–order rate constant for adsorption (min^–1^).

The pseudo–second–order equation is expressed as [66]:

$$q_{t}=\frac{k_{2}q_{e}^{2}t}{1+k_{2}q_{e}t}$$ (6)

where k_2_ is the pseudo–second–order rate constant for adsorption (g mg^–1^ min^–1^).

The Elovich equation is expressed as [67]:

$$q_{t}=A+B\ln t$$ (7)

where A and B are the Elovich constants.

The intra–particle diffusion model is expressed as [68]:

$$q_{t}=K_{i}t^{1/2}+C$$ (8)

where K_i_ is the intra–particle diffusion rate constant (g mg^–1^ min^–1/2^), C (mg g^–1^) is a constant dependent on boundary layer thickness, with larger C values obtained for increased boundary layer effects.

These rate constants and correlation coefficients (R^2^) obtained using these models are shown in Table 4.

**Table 4:** Parameters for kinetic models of Th^4+^ biosorption of on GLP.

Ion	Pseudo–first–order model			Pseudo–second–order model		
	K_1_(min^–1^)	q_e_ (mg g^–1^)	R	K_2_ (g mg^–1^ min^–1^)	q_e_ (mg g^–1^)	R
Th^4+^	0.0028	49.104	0.9334	0.004	51.2	0.9968
Ion	Elovich equation			Intra–particle diffusion model		
	A	B	R	C	Ki	R
Th^4+^	27.29	4.603	0.9586	32.763	1.636	0.9707

The R values in Table 4 showed that the pseudo–second–order kinetic model (R > 0.99) fitted the whole biosorption process better than the other models. The q_e_ values obtained from the pseudo–second–order model was in perfect agreement with the experimental values for Th^4+^ biosorption at different initial concentrations. Therefore, the pseudo–second–order model was concluded to predict the kinetic process under experimental condition.

#### Thermodynamic study

The thermodynamic parameters corresponding to biosorption, including changes in Gibbs free energy (*∆G°*), enthalpy (*∆H°*), and entropy (*∆S°*) [69], were calculated using the following equations [70]:

$$\ln K_{D}=\frac{\Delta S^{{}^{\circ}}}{R}-\frac{\Delta H^{{}^{\circ}}}{RT}$$ (9)

where K_D_ (mL g^–1^) is the distribution coefficient. Changes in enthalpy (*∆H°*) and entropy (*∆S°*) were estimated using the following equation [70, 71]:

$$\Delta G^{{}^{\circ}}=\Delta H^{{}^{\circ}}-T\Delta S^{{}^{\circ}}$$ (10)

**Fig 10:**
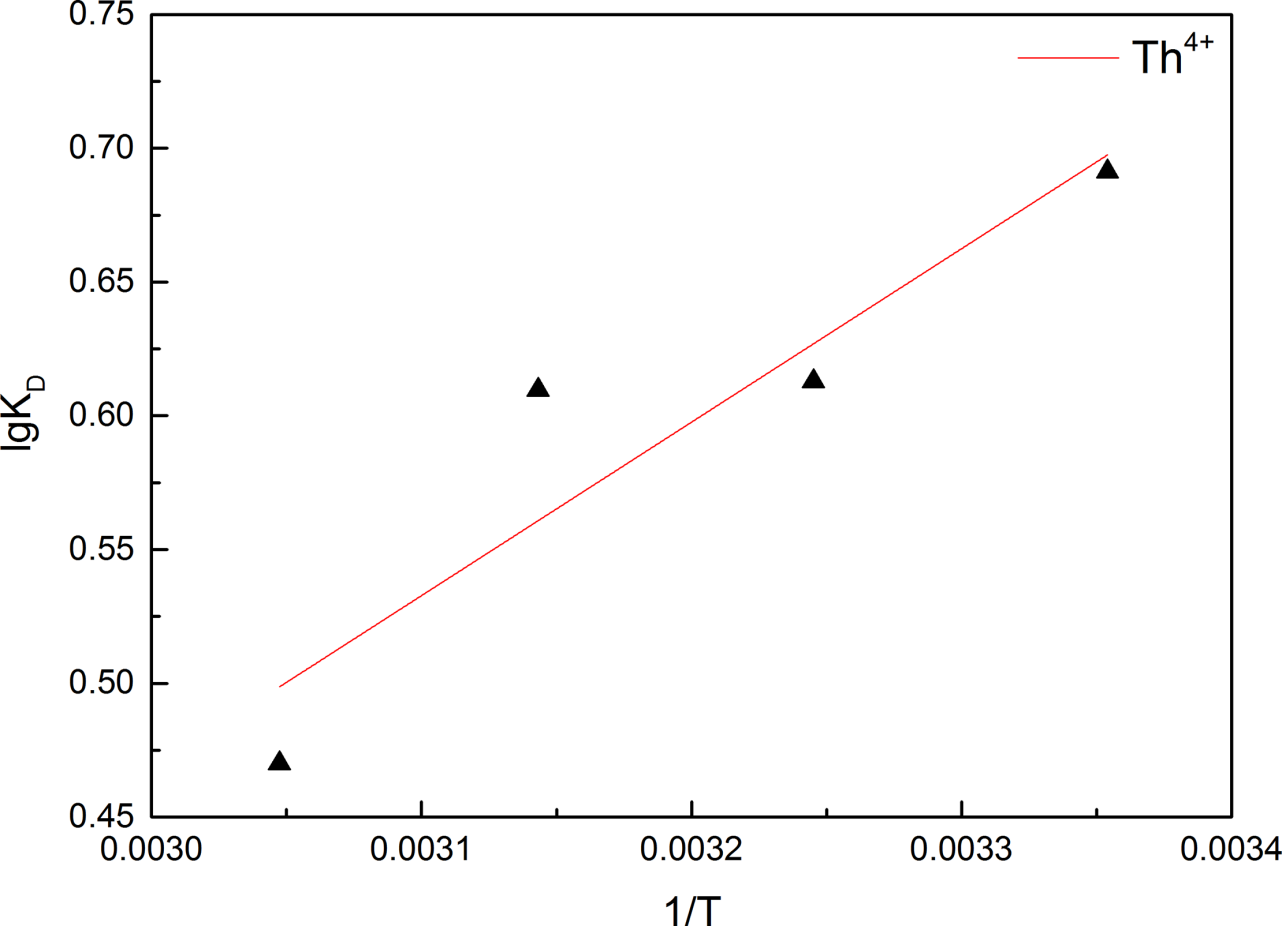
Plot of logK_D_ vs. 1/T for Th^4+^ biosorption on GLP.

*∆H°* and *∆S°* were calculated from the slope and intercept, respectively, of a plot of ln K_D_ vs. *1/T*, as shown in Fig 10. *∆H°*, *∆S°*, and *∆G°* values are shown in Table 5.

**Table 5:** Thermodynamic parameters for Th biosorption on GLP.

Ion	*∆G°* (kJ mol^–1^)				*∆H°* (kJ mol^–1^)	*∆S°* (J mol^–1^ K^–1^)
	25°C	35°C	45°C	55°C		
Th^4+^	–9.051	–9.174	–9.297	–9.420	–5.39	12.28

As shown in Table 5, at different temperatures, Th^4+^ adsorption by GLP had *∆G°* values of less than zero. *∆G°* is used to determine the preference and driving force of a process, with the negative *∆G°* values for Th^4+^ biosorption on GLP indicating a thermodynamically feasible and spontaneous process [14, 72]. The negative *∆H°* values suggested that Th^4+^ complexation with the biosorbent was dominant over other opposing enthalpic factors (such as metal ion dehydration) [73, 74] and confirmed the exothermic nature of the process [75]. The positive *∆S°* values indicated that randomness at the solid/solution interface increased irregularly during Th^4+^ biosorption [76].

#### SEM analysis

**Fig 11:**
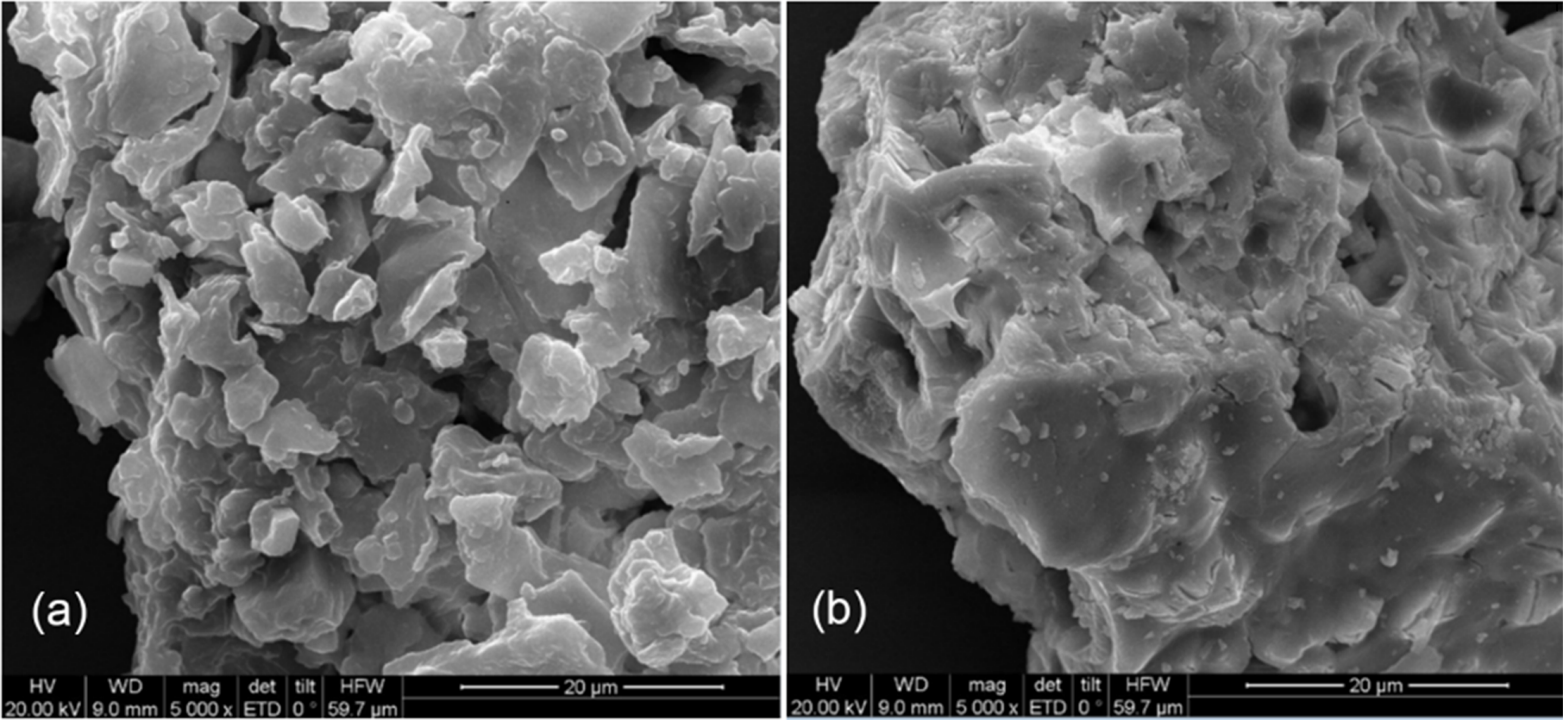
SEM analysis of GLP (a) before biosorption and (b) after biosorption.

Scanning electron microscopy (SEM) has been used to characterize adsorbents and elucidate possible adsorption mechanisms in many studies [77, 78]. In this study, SEM micrographs were obtained before and after Th^4+^ adsorption, as presented in Fig 11(A) and 11(B), respectively. These micrographs clearly showed different deformations and appearances for GLP before and after Th^4+^ adsorption. Fig 11(A) shows native GLP with a porous and irregular surface with a good probability for trapping and adsorbing Th^4+^ in the pores. Fig 11(B) shows Th^4+^–loaded GLP, in which pores are partially blocked with the smooth large particles due to Th^4+^ deposition. Notably, significant adsorption had occurred on the GLP surface.

#### BET analysis

The BET surface area of GLP before biosorption (2.8058 m^2^ g^–1^) was larger than that after biosorption (1.4786 m^2^ g^–1^). The GLP specific surface area had changed slightly after biosorption due to metal ion adsorption, as confirmed by SEM analysis, which showed that the adsorbents had no well–defined holes in their morphology (only a few surface pores) and a small surface area. This confirmed that this process was essentially surface adsorption, not pore adsorption [79].

#### XRD analysis

**Fig 12:**
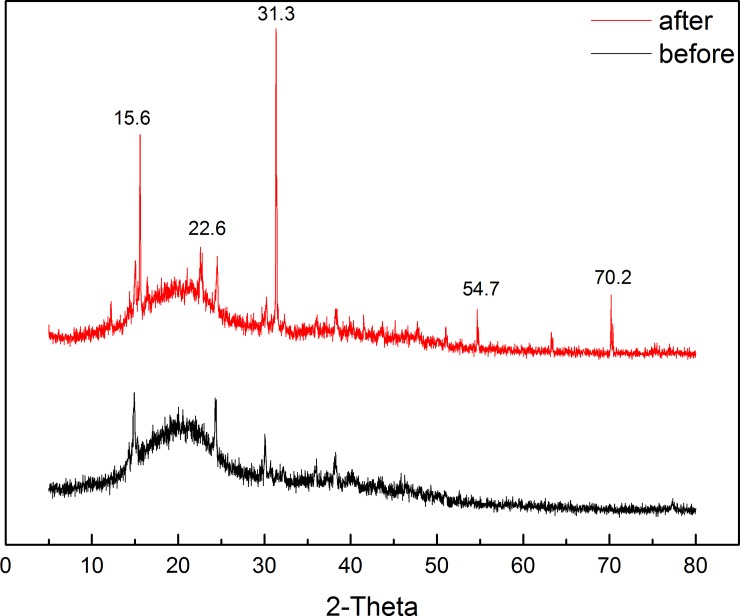
XRD patterns for GLP before and after Th adsorption.

The results of XRD analysis of GLP are shown in Fig 12. The XRD pattern was typical of a cellulosic material, showing main and secondary peaks at 2θ = 24° and 15° corresponding to highly ordered crystalline cellulose and a less ordered polysaccharide structure, respectively. Furthermore, after Th^4+^ biosorption, broad diffraction peaks appeared at 2θ = 15.6°, 22.6°, 31.3°, 54.7°, and 70.2°, which corresponded to diffraction peaks of compounds containing Th ions, indicating that the adsorbent surface contained Th ions.

#### FTIR analysis

**Fig 13:**
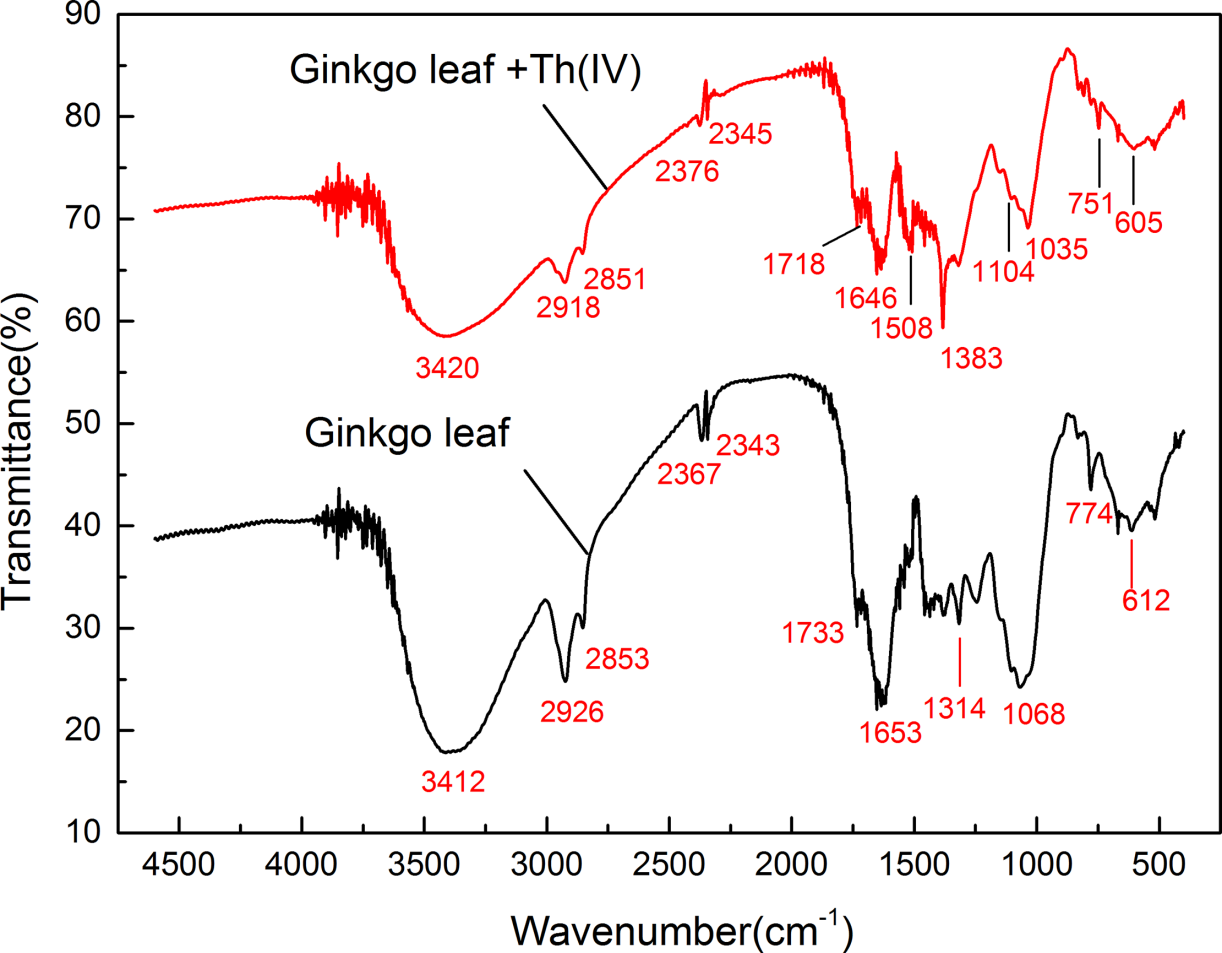
FT–IR spectra of GLP before and after Th^4+^ adsorption.

The pattern of metal ion adsorption on biomaterials is governed by their active groups and bonds [80]. FT–IR spectra of GLP were obtained to determine its functional groups, which play an important role in the analysis of these organic functional groups and the mechanism of Th^4+^ ions adsorbed by the adsorbent. The FT–IR spectra indicated that GLP contains carboxyl and hydroxyl groups, which are able to interact with heavy metal ions in aqueous solution. Fig 13 shows FT–IR spectra of GLP before and after Th^4+^ biosorption, which were complex due to the numerous and various functional groups present. Before biosorption, a broad and intense peak appeared at 3412 cm^–1^, which was assigned to–OH stretching vibrations of alcohols, phenols, and carboxylic acids, as found in pectin, cellulose and lignin [81]. The peak at 2926 cm^–1^ was assigned to symmetric and asymmetric C–H stretching vibrations of–CH_3_, as found in cellulose [82]. The peak at 1733 cm^–1^ corresponded to the C = O stretching vibration in carboxyl (–COOH) and ester (–COOR) groups [83]. The peak observed at 1653 cm^–1^ was attributed to asymmetric C–O stretching vibrations in carboxylate groups (–COO^–^). The peak at 1068 cm^–1^ was attributed to C–OH stretching vibration in alcohols and carboxylic acids.

The FTIR spectrum clearly indicated that carboxyl and hydroxyl groups were abundant in GLP. Therefore, Th^4+^ biosorption on GLP was likely due to electrostatic attraction between these groups and Th^4+^ cations.

After Th adsorption, the sharp band at 1733 cm^–1^ associated with C = O stretching vibrations in GLP was shifted to 1718 cm^–1^ in the complex, suggesting that carbonyl oxygens were involved in coordination with Th^4+^. Changes in peaks at 3412, 2926, 1653, and 1068 cm^–1^ indicated that hydroxyl, carbonyl, and carboxylic groups were also involved in adsorption. Furthermore, sharp peaks were observed at 1383 cm^–1^ and 1035 cm^–1^ that corresponded to the υTh–O band of Th ions, providing direct evidence for Th^4+^ adsorption on GLP under these conditions [84]. These results indicated the functional groups present on the GLP surface and the adsorption mechanism, which is dependent on these functional groups, particularly carboxyl groups.

### Application

Monazite, is among the main ore minerals containing light rare earth elements (REEs) and Th [85]. Although monazite concentrate has long been used for REE and phosphorus extraction, the large amounts of Th and uranium present in monazite slag have not been effectively exploited, and are stored as radioactive waste that is harmful to the environment. Therefore, research into the recovery and utilization of monazite slag to protect the environment from its radioactivity and toxicity is of great significance.

A real sample containing monazite mineral was studied in a batch experiment. The results obtained (Table 6) showed that Th was almost completely adsorbed (90.4%) from the solution, in contrast to the much lower adsorption rate of other metals. These phenomena demonstrated that GLP can be successfully applied as a biosorbent to treat monazite mineral leaching solution containing high radioactive Th ions, and also hinted that Th can be removed from monazite slag at higher concentrations. This is an important development for protecting the environment from radioactive Th ions.

**Table 6:** Percentage biosorption of different ions by GLP from monazite cake dissolved in nitric acid.

Metal ion	Concentration of dissolved metal ions [86] in pH 4 HNO_3_ before biosorption (mg L^–1^)	Biosorption (%)^a^
Th^4+^	121.6	90.4±0.4
UO_2_^2+^	2.3	4.2±0.9
Y^3+^	11.2	2.1±0.1
La^3+^	247.4	2.8±0.3
Ce^3+^	522.4	4.3±1.2
Pr^3+^	58.8	3.3±0.3
Nd^3+^	174.8	2.4±1.4
Sm^3+^	35.4	5.7±1.9
Gd^3+^	16.4	3.0±1.9
Pb^2+^	3	23.5±1.0
Ca^2+^	6.2	12.2±1.6

^**a**^Average values for three independent adsorption experiments; precision corresponds to ± σ_n-1_, where σ_n-1_ is the standard deviation of the mean.

## Conclusions

In summary, we have demonstrated the preparation of three low–cost biosorbents, namely, ginkgo leaf powder, walnut shell powder, and grapefruit peel powder, and their biosorption of lanthanides (La^3+^, Ce^3+^, Pr^3+^, Nd^3+^, Sm^3+^, Eu^3+^, Gd^3+^, Yb^3+^, and Lu^3+^), actinides (UO_2_^2+^ and Th^4+^), transition metal ions (Y^3+^, Co^2+^, Zn^2+^, and Ni^2+^) and an alkali earth metal (Sr^2+^). Compared with other metal cations, excellent lanthanide and actinide adsorption efficiencies were obtained for GLP, WSP, and GFP in single metal systems. Furthermore, these biosorbents, especially GLP, exhibited a noticeable selectivity for Th^4+^, a representative actinide, over lanthanides, transition metal ions, and alkali earth metals under acidic conditions in a mixed–ion aqueous solution. In biosorption studies, effective Th^4+^ biosorption by GLP was achieved under the following conditions: pH 4; contact time, 120 min; biosorbent concentration, 2 g L^–1^; particle size, 150–200 mesh; initial Th concentration, 100 mg L^–1^; temperature, 298 K. Biosorption followed the Langmuir adsorption isotherm model, with a maximum capacity of 103.8 mg g^–1^. Th^4+^ biosorption from aqueous solution by GLP obeyed pseudo–second–order kinetics. Thermodynamic calculations indicated the feasible, exothermic, and spontaneous nature of the biosorption process. More importantly, the good sorption efficiency Th^4+^ of GLP allowed its use in water samples containing monazite mineral, which is of great significance for the recovery and utilization of radioactive contaminants containing Th ions to protect the environment from their radioactivity and toxicity.
